# Organophosphate Pesticide Exposures, Nitric Oxide Synthase Gene Variants, and Gene–Pesticide Interactions in a Case–Control Study of Parkinson’s Disease, California (USA)

**DOI:** 10.1289/ehp.1408976

**Published:** 2015-09-18

**Authors:** Kimberly C. Paul, Janet S. Sinsheimer, Shannon L. Rhodes, Myles Cockburn, Jeff Bronstein, Beate Ritz

**Affiliations:** 1 Department of Epidemiology, Fielding School of Public Health, University of California at Los Angeles (UCLA), Los Angeles, California, USA; 2 Department of Human Genetics, and; 3 Department of Biomathematics, David Geffen School of Medicine, UCLA, Los Angeles, California, USA; 4 Department of Biostatistics, Fielding School of Public Health, UCLA, Los Angeles, California, USA; 5 Department of Preventive Medicine, Keck School of Medicine, University of Southern California, Los Angeles, California, USA; 6 Department of Neurology, School of Medicine, UCLA, Los Angeles, California, USA; 7 Department of Environmental Health Sciences, Fielding School of Public Health, UCLA, Los Angeles, California, USA

## Abstract

**Background:**

Nitric oxide synthase (NOS) genes are candidates for Parkinson’s disease (PD) because NOS enzymes produce nitric oxide (NO), a pro-oxidant that can damage neurons. Widely used organophosphate (OP) pesticides can induce oxidative stress and are reported to increase PD risk. Additionally, two single nucleotide polymorphisms (SNPs) from the PON1 (paraoxonase 1) gene influence the ability to metabolize OPs.

**Objective:**

Here, we investigated contributions of NOS genes and OP pesticides to PD risk, controlling for PON1 status.

**Methods:**

In 357 incident PD cases and 495 population controls, we investigated eight NOS SNPs and interactions with both household and ambient agricultural OP exposures assessed with geographic information system (GIS).

**Results:**

In comparing PD in homozygous variant carriers of NOS2A rs1060826 versus homozygous wild-type or heterozygotes, we estimate an adjusted odds ratio (OR) of 1.51 (95% CI: 0.95, 2.41). When considering interactions between NOS1 rs2682826 and OP exposure from household use, the OR for frequent OP use alone was 1.30 (95% CI: 0.72, 2.34) and for the CT+TT genotype alone was 0.89 (95% CI: 0.58, 1.39), and for frequent OP use combined with the CT+TT genotype the OR was 2.84 (95% CI: 1.49, 5.40) (interaction p-value 0.04). Similar results were seen for ambient OP exposure. Interactions between OP exposure and three other NOS1 SNPs and a genetic risk score combining all NOS1 SNPs reached statistical significance.

**Conclusions:**

We found that OP pesticides were more strongly associated with PD among participants with variant genotypes in NOS1, consistent with the importance of oxidative stress-inducing mechanisms. Our data provide evidence for NOS1 modifying PD risk in OP exposed populations.

**Citation:**

Paul KC, Sinsheimer JS, Rhodes SL, Cockburn M, Bronstein J, Ritz B. 2016. Organophosphate pesticide exposures, nitric oxide synthase gene variants, and gene–pesticide interactions in a case–control study of Parkinson’s disease, California (USA). Environ Health Perspect 124:570–577; http://dx.doi.org/10.1289/ehp.1408976

## Introduction

Parkinson’s disease (PD) is a neurodegenerative disorder characterized by the progressive depletion of dopaminergic neurons in the substantia nigra of the brain. Both genetic and environmental factors alone can cause Parkinsonism, as seen with rare mutations in several genes linked to familial PD (Schiesling et al. 2008) and exposure to the toxic metabolite of MPTP (1-methyl-4-phenyl-1,2,3,6-tetrahydropyridine) (Langston 1985). Idiopathic PD, however, is believed to result from multiple etiologies, most of which likely require not only exposure to environmental toxins but also an underlying genetic susceptibility (Schapira 2006). Several major molecular pathways are implicated in PD pathogenesis, including mitochondrial dysfunction resulting from or in oxidative/nitrosative stress (Dauer and Przedborski 2003), inspiring investigators to focus on reactive oxygen or nitrogen species (ROS/RNS) such as nitric oxide (NO) or certain pesticide metabolites (Ryan et al. 2013). Although a number of genetic variants and environmental factors have been consistently implicated in PD etiology, rarely have reported gene–environment interactions been replicated, including those for nitric oxide synthase gene variants and pesticide exposures.

NO, a chemical messenger and free radical by-product of reactions catalyzed by nitric oxide synthase (NOS) enzymes, is essential for numerous physiologic processes, including neurotransmission, but is also a pro-oxidant capable of contributing to oxidative/nitrosative stress and damaging an array of cell types, including dopaminergic neurons (Kavya et al. 2006). Three genes encode NOS enzymes: *NOS1* on chromosome (chr) 12 encodes neuronal NOS (nNOS), *NOS2A* on chr 17 encodes inducible NOS (iNOS), and *NOS3* on chr 5 encodes endothelial NOS (eNOS). *NOS1* and *NOS2A* are of particular interest in PD because of their expression in the brain (Licinio et al. 1999). Several single nucleotide polymorphisms (SNPs) in the *NOS1* and *NOS2A* genes have previously been linked to PD risk, but few reports have implicated the same SNPs (Hague et al. 2004; Hancock et al. 2008; Huerta et al. 2007; Levecque et al. 2003; Schulte et al. 2006). Although the functionality of these SNPs is still unknown and the epidemiologic evidence inconclusive, much stronger support for an involvement of *NOS* in neurotoxicity is provided by laboratory studies. In animal models, inhibition of nNOS prevents MPTP-induced Parkinsonism in both baboons and mice (Hantraye et al. 1996; Schulz et al. 1995), and MPTP-induced neuronal damage is diminished in mice lacking either the *NOS1* or the *NOS2A* gene (Liberatore et al. 1999; Przedborski et al. 1996). Postmortem studies also found higher levels of NO in the nigrostriatal region in PD brains (Hunot et al. 1996).

Organophosphate (OP) pesticides, commonly used agriculturally and until recently in households, have long been investigated in relation to PD, not only because of their neurotoxicity through action on acetylcholinesterase, their primary target, but also their ability to induce oxidative stress through increased production of reactive oxygen species (Bagchi et al. 1995; Lukaszewicz-Hussain 2010). With evidence that both NO and pesticide exposures are contributing to neuronal damage through the same pathways, we speculate that they may act synergistically to increase PD risk. For example, OP-induced oxidative stress alone has the potential to lead to mitochondrial complex 1 dysfunction and as a result further generation of superoxides; however, superoxides readily react with NO to form peroxynitrite (NO_3_^–^), a more potent toxicant able to irreversibly inhibit mitochondrial respiration (Dauer and Przedborski 2003; Kavya et al. 2006; Lukaszewicz-Hussain 2010). Adding more complexity, variations in two functional SNPs of the *PON1* (*paraoxonase 1*) gene are known to influence the ability to metabolize and detoxify OPs and influence PD risk (Lee et al. 2013). Statistical interactions between *NOS1* SNPs and home pesticide use in PD were first seen in a North American family study (Hancock et al. 2008). Here, we attempt to replicate this reported finding for household pesticide use, examining interactions with household pesticide exposures, and also to contribute new information about the interaction with OP pesticides specifically, both from household use and ambient exposure to agricultural pesticides, and with other *NOS1* genetic variants, while also taking into account increased susceptibility due to *PON1* status.

## Materials and Methods

All procedures described were approved by the University of California at Los Angeles (UCLA) Human Subjects Committee, and written informed consent was obtained from all participants.

*Participant recruitment.* We enrolled incident PD patients along with population-based controls between January 2001 and December 2010 from three highly agricultural central California counties (Kern, Tulare, Fresno) known for the high use of agricultural pesticides. Detailed participant recruitment (Costello et al. 2009; Wang et al. 2011) and case definition criteria (Jacob et al. 2010; Kang et al. 2005) have been previously described and published.

Briefly, of the 1,167 PD patients initially identified through large medical groups, neurologists, and public service announcements, 604 did not meet eligibility criteria for the following reasons: 397 were not diagnosed with PD within 3 years before recruitment, 134 lived outside the tri-counties, and 73 did not have PD. From the 563 potential cases, 90 could not be examined by our movement disorder specialist (J.B.), 56 declined or moved away, 34 became too ill or died before the scheduled appointment; of 473 examined by us (J.B.), 94 did not meet published criteria for idiopathic PD (Hughes et al. 1992), an additional 13 were reclassified as not having PD during follow-up (Ritz et al. 2012), and 6 participants withdrew between examination and interview. Of the remaining 360 cases, 357 provided information and biologic samples necessary for inclusion in at least one of our analyses.

To be eligible as population-based controls, participants must have been > 35 years of age, having lived within one of the three counties for at least 5 years before enrollment, and not have a diagnosis of PD. We identified potentially eligible population-based controls from the same tri-county area initially through both Medicare enrollee lists (2001) and publicly available residential tax-collector records (2001–2010) (Kern, Fresno, and Tulare County Tax Assessor), and after 2001 only through residential tax-collector records. We used two sampling strategies to increase enrollment success and representativeness of the source population: *a*) random selection from the Medicare enrollee lists and of residential parcels (identified from the tax-collector records) followed by mail or phone enrollment, and *b*) random selection of clustered households (five per cluster, identified through the tax-collector records) we visited in person to enroll eligible controls; these enrollment methods have been described in more detail previously (Costello et al. 2009; Wang et al. 2011).

From the first sampling method, we contacted 1,212 potentially eligible controls. Of these individuals, 457 were ineligible: 409 were < 35 years of age, 44 were too ill to participate, and 4 resided primarily outside the study area. Of the 755 eligible population controls, 409 declined participation, were too ill, or moved before an interview was possible; resulting in the enrollment of 346 population controls. From the second sampling strategy, 4,756 individuals were screened, of whom 3,515 were ineligible (88% of these were out of the age range) and 634 of the eligible controls declined participation; 607 population controls were enrolled, but 183 of them completed only an abbreviated interview and did not contribute all data needed for this analysis. Additionally, an early mailing (for which the number of eligible participants who declined was not known) produced 62 controls. Of the 832 recruited controls, 337 were excluded because they lacked *NOS* genotyping data. Thus, in total only 495 controls provided information and biologic samples necessary for inclusion in at least one of the analyses, 333 originating from the first control recruitment effort.

*Pesticide exposure assessment.* Cases and controls were interviewed by telephone to obtain information on demographic characteristics, risk factors, and included detailed questions on home pesticide use and lifetime occupational and residential histories. During the interview, participants provided information on chemical use in the home, lawn, or garden. More detail on this exposure assessment has been published (Narayan et al. 2013). Briefly, participants were asked to recall names of chemicals or products if possible, or partial product names, manufacturer names (e.g., Raid), targets (e.g., weed control, plant disease, ants, spiders), or formulation of products (e.g., liquid, granules, bait). These interview data were supplemented with information about ingredients from the California Department of Pesticide Regulation (CDPR) product label database (CDPR 2013b). The active ingredient (chemical contributing the largest percentage to a product’s composition) was then categorized into chemical classes, again using the CDPR product label database. Interviewers additionally asked about frequency of use [none or rarely (once a year or less), sometimes (2–11 times a year), or regularly (more than once a month)] during four different periods: young adult (16–24 years), adult (25 to < 45 years), middle age (45 to < 65 years), and senior (≥ 65 years). Only use by the participant themselves was considered.

We assessed lifetime home pesticide use by calculating a weighted average frequency of use (Narayan et al. 2013). For each pesticide class we multiplied the midpoint of the frequency category by years in each age period up to 10 years before index date (date of diagnosis or interview), summed across age periods, and divided by the total number of years between ages 16 and 10 years before index date. Those with an average frequency of use of any reported pesticide class above or equal to the pesticide class–specific median found in exposed controls were considered “frequent users” of any pesticide and those with an average frequency of use below the median for all pesticides as “occasional users” for the “any household pesticide use” exposure assessment. For household use of OPs, those with an average frequency above or equal to the OP use median found in exposed controls were considered “frequent users” of OPs, and those with an average use below the median to all pesticides as “occasional users.” We then classified participants in mutually exclusive groups, as “frequent users” of OP pesticides, as described above, “frequent users” of other non-OP pesticides, those who did not frequently use OPs but did use other pesticides frequently, and “occasional users” of pesticides; in primary analysis for household OP use, we excluded “frequent users” of other non-OP pesticides, only comparing “frequent users” of OP pesticides to “occasional users,” comparisons using “frequent users” of non-OPs were included in secondary analyses.

Ambient pesticide exposure resulting from commercial applications to agricultural crops was estimated using a geographic information system (GIS)–based computer model, which links geocoded lifetime residential and occupational address histories of participants; California state-mandated pesticide use report (CA-PUR) data (CDPR 2013a), which include information on all agricultural pesticide applications and the date, location, and amount applied; and land use surveys from California’s Department of Water Resources (CDWR 2013), which provides the location of specific crops. We provide a brief description here, and a more detailed and technical discussion of the GIS method has been published (Cockburn et al. 2011). For all pesticides, we summed the pounds of chemical applied per year per acre within a 500-m radius buffer of each address. For each participant, we then calculated a study period average for each chemical from 1974 to 10 years before the participant’s index year by summing the year-specific averages and dividing that sum by the total number of years in the relevant time period. If a participant was missing geocode location information for any given year, we used simple imputation, substituting the individual’s average value from their recorded years. CA-PUR data indicated that the study population was exposed to 36 different chemicals classified as OPs based on information from CDPR and the pesticide action network (PAN) pesticide database (Kegley et al. 2014) (see Supplemental Material, Table S1, for a complete list). Exposures over the same time period at both residential and occupational addresses were included, and each participant could have been exposed at both locations, only one, or neither. We dichotomized exposure to each of the individual OP chemicals based on each chemical’s median level in exposed controls, and then summed the number of OP chemicals that each participant was exposed to above the median, counting chemical exposures from both residence and occupation. We then classified OP exposure based on the exposure distribution of the OP sum from the controls in the following manner: high exposure, exposed to > 11 OP chemicals (top quartile in exposed controls), and none/low exposure: 0–11 OP chemicals.

In addition to using CA-PUR data to estimate ambient exposure at each occupational location, direct occupational exposure was derived from a job exposure matrix (JEM), where participants’ level of exposure was estimated for each reported occupation (Liew et al. 2014). However, JEM-based occupational exposure was not used for our primary analysis because exposures to the specific pesticides of interest here, OPs, could not be estimated, but was included as a covariate to control for other sources of pesticide exposure.

*SNP selection and genotyping methods.* Altogether eight SNPs from *NOS1* and *NOS2A* were selected: *NOS1* rs2682826 and rs1047735 and *NOS2A* rs1060826 based on previous PD research (Hague et al. 2004; Hancock et al. 2008; Levecque et al. 2003) and *NOS1* rs3741475, rs3741480, and rs816353 and *NOS2A* rs2297518 and rs3730013 to optimize gene coverage.

Participants provided blood or saliva samples for genetic analyses, which were stored and processed at the UCLA Biologic Specimen Core Facility. Several collaborative research projects performed genotyping of our samples for these SNPs; this led to a different number of participants with data available for each SNP. *NOS1* SNPs rs1047735, rs2682826, and rs3741475 and *NOS2A* SNPs rs1060826, rs2297518, and rs3730013 genotyping was conducted at Stanford Human Genome Center; PCR (polymerase chain reaction) assays were conducted with TaqMan Universal Master Mix (Applied Biosystems), primers and probes were designed based on the NCBI (National Center for Biotechnology Information) DNA sequence and purchased from ABI (Applied Biosystems). Fluorescence data files from each plate were analyzed by automated allele calling software (ABI Prism 7900 HT Sequence Detection System 2.1). Fill-in genotyping for additional cases and controls recruited later in the study for each of these SNPs except rs3741475 was performed at the UCLA Genomics Core Facility using the Applied Biosystems SNPlex array (Tobler et al. 2005). *NOS1* rs3741480 and rs816353 genotyping was performed at the University of Washington’s SF Functional Genomics and Bioinformatics Core Laboratory using the Fluidigm BioMark HD system (Fluidigm Corporation). All SNPs had a call rate > 98.5% except for rs1047735 (97%), rs3741480 (93%), and rs816353 (93%). Additionally, *PON1* genotyping was conducted at the UCLA Genomics Core using pyrosequencing for L55M (rs854560), and for Q192R (rs662) using the Fluidigm BioMark HD system at the University of Washington. *PON1* metabolizing status was based on published report (O’Leary et al. 2005); briefly, “slower” metabolizers are considered those with an MM genotype at L55M and QQ or QR at Q192R; other genotypes are considered “faster” metabolizers.

To safeguard against systematic genotype errors due to using different genotyping centers for fill-in genotyping of *NOS1* SNPs rs1047735 and rs2682826, and *NOS2A* SNPs rs1060826, rs2297518, and rs3730013, 97 participants were included in both genotyping experiments; they provided an interlaboratory genotype call rate concordance of 99.8% (one discordant call at rs2682826 for 1 participant). This is in addition to the 5–10% duplicate samples included in each individual experiment used to confirm quality genotyping. Additionally, for each of the five SNPs, we used logistic regression to examine whether genotype could predict the centers where genotyping was performed, assuming an allelic model (comparing the minor allele to the major allele), and using the control population only; we found no statistically significant associations by center, suggesting no systemic error by center (data not shown).

NOS1 *genetic risk score.* We created a genetic risk score (GRS) based on the five *NOS1* SNPs genotyped. The score counts the minor alleles, such that participants homozygous for the minor allele received a 2, heterozygous participants a 1, and those homozygous wild type a 0, at each locus, for a total range of 0–10. In sensitivity analyses, we examined an alternate GRS based on three *NOS1* SNPs only (rs2682826, rs1047735, and rs3741480, total range 0–6), excluding rs816353, as it is in moderate LD (linkage disequilibrium) (*r* ^2^ = 0.6) with rs1047735 and was not in Hardy–Weinberg equilibrium, and rs3741475, as it is in moderate LD (*r* ^2^ = 0.6) with rs2682826.

*Statistical methods.* We examined Hardy–Weinberg equilibrium in control participants for all polymorphisms using a chi-square test, and checked LD between each SNP. We used unconditional logistic regression to calculate odds ratios (ORs) and 95% confidence intervals (CIs) for SNP marginal effects, assuming a recessive genetic model (homozygous for the minor allele vs. any major allele) for *NOS2A* rs1060826 and a dominant model (any minor allele vs. homozygous for the major allele) for *NOS1* rs1047735 and rs2682826, to compare with prior report (Levecque et al. 2003). Because previous model selection was not based on functional significance, for these SNPs and all other SNPs for which we had no *a priori* genetic hypotheses, we additionally assumed an additive genetic model (where each copy of the variant allele increases the risk by the same amount). We also adjusted for potential confounders including sex, age (continuous), cigarette smoking status (ever/never), European ancestry (yes, exclusively European ancestry/no, any non-European ancestry), education (< 12 years, 12 years, > 12 years), and *PON1* metabolizing status [“faster” or “slower” metabolizers (O’Leary et al. 2005)].

Gene–environment interactions were assessed with *NOS1* rs2682826 and pesticide use due to previous report (Hancock et al. 2008) during primary analysis. Statistical interactions were assessed by introducing a multiplicative interaction term (e.g., product term: gene × pesticide) into a logistic model that relied on a dominant genetic model. We also conducted secondary, exploratory analyses, assessing interactions between all other *NOS* SNPs and OP exposure, assuming a dominant genetic model, and between the genetic risk score and OP exposure.

A multiple test correction was not implemented because the SNPs under analyses were selected based on previous research reports that supported associations with PD. Gene–gene interaction analyses between *NOS* SNPs and *PON1* status were not performed, because we did not have an *a priori* hypothesis supporting this relationship, and we had concerns about sufficient power given both the lack of marginal genetic effects and our sample size (≥ 80% power to detect interaction OR ≥ 3.2).

We conducted sensitivity analyses for SNP marginal effects restricting to participants with European ancestry only and adjusting for PD family history (PD in a first degree relative: yes/no). For gene–environment analyses, in sensitivity analysis we mutually adjusted for household OP pesticide use, ambient OP exposure, and occupational exposures derived from our JEM. We also assessed the interaction between household use of non-OP pesticides and NOS rs2682826.

Power calculations were performed using Quanto version 1.2.4 (Gauderman and Morrison 2006), LD using Haploview (Barrett et al. 2005), and all other analyses using SAS 9.3 (SAS Institute Inc.).

## Results

Study participants were predominantly of European ancestry, > 65 years of age, and did not report a family history of PD (Table 1). Cases had a higher proportion of males, never smokers, and slower PON1 metabolizers (Table 1); 121 participants [48 cases (13%) and 73 controls (15%)] were missing *PON1* genotyping and thus PON1 metabolizer status. Cases were more likely to have frequently used household pesticides (OR = 1.69; 95% CI: 1.21, 2.36 for any pesticide use, and OR = 2.05; 95% CI: 1.30, 3.24 for OP use specifically), and to have had high ambient exposure to agricultural OP pesticides (OR = 2.99; 95% CI: 1.92, 4.65; see Supplemental Material, Table S1). All models controlled for age, sex, smoking status, European ancestry, education, and PON1 metabolizer status.

**Table 1:** General characteristics of the study population, *n* = 852.

Characteristic	Cases (*n* = 357)	Controls (*n* = 495)
Age (years) of PD diagnosis [median (range)]	70 (34–88)
Age (years) at interview [median (range)]	72 (37–90)	68 (35–94)
Male sex [*n* (%)]	204 (57)	243 (49)
First degree relative with PD [*n* (%)]
No	304 (85)	450 (91)
Yes	53 (15)	45 (09)
Cigarette smoking [*n* (%)]
Never	188 (53)	227 (46)
Former	150 (42)	221 (45)
Current	19 (05)	47 (09)
European ancestry [*n* (%)]
Yes	288 (81)	441 (89)
No	69 (19)	54 (11)
Education (years) [*n* (%)]
0 to < 12	65 (18)	43 (09)
12	95 (27)	101 (20)
> 12	197 (55)	351 (71)
PON1 metabolizer status [*n* (%)]^*a*^
Faster	264 (85)	376 (89)
Slower	45 (15)	46 (11)
^***a***^121 participants missing PON1 genotypes: 48 cases (13%) and 73 controls (15%).		

The population was in Hardy–Weinberg equilibrium for all SNPs evaluated (*p* > 0.05), except for rs816353 (*p* = 0.02) (Table 2), and the SNPs in each gene were in low to moderate LD with each other (*NOS1 r* ^2^ values ranging from 0.27 to 0.59, for *NOS2A* from < 0.10 to 0.30) (data not shown). For *NOS2A* rs1060826, cases were more likely to have a homozygous variant (OR = 1.56; 95% CI 1.01, 2.38 without PON1 adjustment and OR = 1.51; 95% CI: 0.95, 2.41 with PON1 adjustment) (Table 2). We did not find any other SNPs to be significantly associated with PD, aside from *NOS1* rs1047735 based on an additive model without adjustment for PON1; however, we did not detect an association with the *a priori* selected dominant model (Levecque et al. 2003) (Table 2). When we restricted analyses to participants of European ancestry only, results did not change (data not shown).

**Table 2:** Marginal estimates (ORs and 95% CIs) for genetic variation in *NOS1* and *NOS2A *SNPs in association with PD, assuming an additive genetic model unless otherwise specified.

SNP/genotype	Cases [*n* (%)]	Controls [*n* (%)]	Model 1: no *PON1* adjustment		Model 2^*a*^: *PON1* adjustment		SNP HWE *p*-value^*c*^
			Adjusted OR^*b*^ (95% CI)	*p*-Value	Adjusted OR^*b*^ (95% CI)	*p*-Value	
*NOS1* rs1047735^*d*^
CC	155 (45)	211 (51)	1.00		1.00
CT	143 (41)	176 (42)	1.28 (1.02, 1.60)		1.18 (0.92, 1.52)
TT	49 (14)	30 (07)	1.63 (1.04, 2.55)	0.04	1.39 (0.84, 2.29)	0.20
CT/TT vs. CC			1.20 (0.90, 1.61)	0.22	1.07 (0.78, 1.47)	0.68	0.41
*NOS1* rs2682826^*d*^
CC	178 (50)	231 (51)	1.00		1.00
CT	154 (43)	198 (44)	1.08 (0.85, 1.36)		1.13 (0.87, 1.46)
TT	24 (07)	26 (06)	1.16 (0.72, 1.85)	0.54	1.28 (0.76, 2.14)	0.36
CT/TT vs. CC			1.05 (0.79, 1.39)	0.74	1.11 (0.81, 1.52)	0.51	0.06
*NOS1* rs3741475
CC	167 (64)	165 (64)	1.00		1.00
CT	84 (32)	84 (33)	1.07 (0.78, 1.48)		0.99 (0.69, 1.41)
TT	11 (04)	7 (03)	1.15 (0.61, 2.18)	0.66	0.97 (0.47, 2.00)	0.94	0.34
*NOS1* rs3741480
TT	93 (33)	138 (33)	1.00		1.00
TC	130 (46)	215 (51)	0.90 (0.72, 1.12)		0.90 (0.72, 1.12)
CC	58 (21)	69 (16)	0.81 (0.52, 1.27)	0.36	0.81 (0.52, 1.27)	0.36	0.33
*NOS1* rs816353
GG	89 (32)	143 (34)	1.00		1.00
TG	141 (50)	225 (53)	1.21 (0.96, 1.53)		1.21 (0.96, 1.53)
TT	51 (18)	54 (13)	1.47 (0.93, 2.33)	0.10	1.47 (0.92, 2.33)	0.10	0.02
*NOS2A* rs1060826^*e*^
GG	129 (36)	179 (42)	1.00		1.00
AG	170 (48)	204 (48)	1.28 (1.03, 1.59)		1.26 (0.99, 1.59)
AA	57 (16)	46 (11)	1.63 (1.06, 2.52)	0.03	1.58 (0.99, 2.53)	0.06
AA vs. GG/AG			1.56 (1.01, 2.38)	0.04	1.51 (0.95, 2.41)	0.08	0.28
*NOS2A* rs2297518
GG	238 (67)	268 (62)	1.00		1.00
AG	108 (30)	138 (32)	0.84 (0.65, 1.08)		0.78 (0.59, 1.03)
AA	9 (03)	25 (06)	0.70 (0.42, 1.17)	0.18	0.61 (0.35, 1.06)	0.08	0.20
*NOS2A* rs3730013
CC	156 (44)	193 (46)	1.00		1.00
CT	161 (45)	190 (45)	0.99 (0.79, 1.24)		1.02 (0.80, 1.30)
TT	39 (11)	39 (09)	0.99 (0.63, 1.55)	0.96	1.04 (0.64, 1.69)	0.88	0.43
^***a***^121 participants missing *PON1* genotype: 48 cases (13%) and 73 controls (15%). ^***b***^Additionally adjusted for age (continuous), sex, ever smoked, education, and European ancestry indicator. ^***c***^Hardy–Weinberg equilibrium *p*-value based on control population only. ^***d***^Additionally assumed dominant genetic model due to prior report. ^***e***^Additionally assumed recessive genetic model due to prior report.							

Investigating *NOS1* rs2682826 and any household pesticide, we estimated a nonsignificant interaction based on the *p*-value of the product term (*p* = 0.18; see Supplemental Material, Table S2). When we limited to household OP use specifically (excluding those with frequent use on non-OP pesticides), the product term reached statistical significance (*p* = 0.04); the genetic variant in occasional users of household pesticides did not contribute to an increased risk of PD, in contrast to frequent OP users, where OP exposed variant T-allele carriers were at increased risk compared to the wild-type (OR_CC + OP use_ = 1.30; 95% CI: 0.72, 2.34 vs. OR_CT/TT + OP use_ = 2.84; 95% CI: 1.49, 5.40; Table 3; see Supplemental Material, Figure S1). When we limited household pesticide use to only non-OP pesticides (excluding those with frequent use of OP pesticides), we did not see a significant interaction (interaction *p*-value = 0.66; see Supplemental Material, Table S2). Results for ambient OP exposures were similar to those seen with household OP use; again the genetic variant in those with no/low ambient OP exposure did not influence PD risk (OR_CC_ = 0.99; 95% CI: 0.70, 1.40), whereas high ambient OP exposure in the wild-type population was associated with an increased PD risk (OR_CC + OP exp_ = 2.42; 95% CI: 1.27, 4.61), and those with a variant allele highly exposed to ambient OPs were at the highest risk (OR_CT/TT + OP exp_ = 4.83; 95% CI: 2.39, 9.73) (Table 3) (interaction *p*-value = 0.15).

**Table 3:** Interaction, main, and joint effect estimates for *NOS1* SNPs and OP exposure in association with PD.

SNP	Major/minor allele	Exposure category	Homozygous wild-type			Variant allele carrier			*p* for Interaction
			Cases/controls	Adj OR^*a*^(95% CI)	*p*-Value	Cases/controls	Adj OR^*a*^(95% CI)	*p*-Value	
Household OP use^*b*^
*NOS1* rs2682826	C/T	Occasional use	81/109	1.00 (reference)		70/104	0.89 (0.58, 1.39)	0.62
		Frequent use	32/39	1.30 (0.72, 2.34)	0.39	41/22	2.84 (1.49, 5.40)	0.002	0.04
*NOS1* rs1047735	C/T	Occasional use	82/107	1.00 (reference)		64/96	0.82 (0.52, 1.28)	0.39
		Frequent use	33/33	1.62 (0.88, 2.98)	0.12	38/23	2.31 (1.22, 4.37)	0.01	0.21
*NOS1* rs816353	G/T	Occasional use	51/80	1.00 (reference)		85/154	0.86 (0.55, 1.34)	0.5
		Frequent use	19/27	1.22 (0.60, 2.51)	0.58	49/40	2.01 (1.12, 3.59)	0.02	0.14
*NOS1* rs3741480	T/C	Occasional use	83/161	1.00 (reference)		53/74	0.73 (0.46, 1.14)	0.16
		Frequent use	53/40	0.93 (0.44, 1.99)	0.85	15/26	1.90 (1.06, 3.41)	0.03	0.02
*NOS1* rs3741475	C/T	Occasional use	86/84	1.00 (reference)		33/43	0.70 (0.39, 1.25)	0.23
		Frequent use	32/25	1.59 (0.83, 3.07)	0.17	21/6	3.46 (1.28, 9.37)	0.01	0.07
Ambient OP exposure^*c*^
*NOS1* rs2682826	C/T	None/low	117/169	1.00 (reference)		116/162	0.99 (0.70, 1.40)	0.96
		High	33/18	2.42 (1.27, 4.61)	0.01	43/12	4.83 (2.39, 9.73)	< 0.0001	0.15
*NOS1* rs1047735	C/T	None/low	114/155	1.00 (reference)		112/156	0.93 (0.65, 1.32)	0.67
		High	32/19	2.07 (1.09, 3.91)	0.03	42/10	5.42 (2.54, 11.52)	< 0.0001	0.04
*NOS1* rs816353	G/T	None/low	72/118	1.00 (reference)		142/245	0.95 (0.66, 1.36)	0.77
		High	17/17	1.59 (0.76, 3.32)	0.22	50/19	4.24 (2.30, 7.83)	< 0.0001	0.03
*NOS1* rs3741480	T/C	None/low	139/250	1.00 (reference)		75/112	0.83 (0.57, 1.19)	0.3
		High	49/19	1.43 (0.69, 2.96)	0.33	18/18	3.78 (2.04, 6.99)	< 0.0001	0.01
*NOS1* rs3741475	C/T	None/low	111/114	1.00 (reference)		56/64	0.86 (0.54, 1.36)	0.52
		High	38/14	2.93 (1.48, 5.80)	0.002	25/5	4.52 (1.61, 12.64)	0.004	0.36
^***a***^Adjusted (Adj) for age (continuous), sex, ever-smoked, European ancestry, education, and PON1 status. ^***b***^Participants with an average frequency of household OP use per year during ages 16 to < 10 years before index age that was at or above the median average use in exposed controls were assigned to the “Frequent Use” category. Those in the “Occasional Use” category had an average frequency of use per year during ages 16 to < 10 years before index age that was below the median for any household pesticide (excluded subjects who did not frequently use OPs but frequently used other pesticides). ^***c***^Ambient pesticide exposure, counting total number of OPs exposed to (above the median level seen in exposed controls) at both occupation and residence, from 1974 (year of CA-PUR implementation) to 10 years before diagnosis or interview. Cut point was based on top quartile in exposed controls.									

In secondary, exploratory analysis, we detected three other significant statistical interactions between *NOS1* SNPs rs1047735, rs816353, and rs3741480, and ambient OP exposure (Table 3). For each SNP, we detected a moderate pesticide association in homozygous wild-type carriers, comparing those highly exposed to ambient OPs with a wild-type genotype with those who had no/low exposure and a wild-type genotype [ORs range from 1.43 (95% CI: 0.69, 2.96) to 2.07 (95% CI: 1.09, 3.91] (Table 3); whereas highly exposed variant allele carriers were at the highest risk when compared with those with no/low exposure and a wild-type genotype [ORs range from 3.78 (95% CI: 2.04, 6.99) to 5.42 (95% CI: 2.54, 11.52)] (Table 3). Similar trends are seen also with the household OP use, though only the product term with rs3741480 reached statistical significance; we detected no increase to moderate nonsignificant increases in risk when comparing frequent use of OP pesticides to occasional use in wild-type carriers [ORs range from 0.93 (95% CI: 0.44, 1.99) to 1.62 (95% CI: 0.88, 2.98)] (Table 3), and the highest risk was found in variant allele carriers who frequently used OP pesticides compared with wild-type occasional users [ORs range from 1.90 (95% CI: 1.06, 3.41) to 2.31 (95% CI: 1.22, 4.37)] (Table 3). We also detected interactions using the GRS, again based on the product term between the GRS, which we treated as a linear variable, and the pesticide exposure indicators (*p*-values for interaction ranged from 0.01 to 0.09; see Supplemental Material, Table S3). For example, for the five SNP *NOS1* GRS (range, 0–10 variant alleles) and ambient OP exposure, no significant risk increase was detected per additional variant allele copies in those with no or low exposure (OR_per 1 variant allele_ = 1.04; 95% CI: 0.97, 1.12), as seen with each individual SNP, whereas in those highly exposed to ambient OP exposure, there was a significant increase in risk per each additional variant allele copy (OR_per 1 variant allele + OP exp_ = 1.90; 95% CI: 1.04, 3.43).

Investigating *NOS2* SNPs, we did not detect any marginal associations except for *NOS2A* rs1060826 (Table 2) or significant interactions with pesticide exposure, based on the *p*-value of the product term (data not shown). Mutually adjusting for household OP pesticide use, ambient OP exposures, ambient maneb and paraquat exposures, occupational exposures to pesticides (JEM), and other *NOS* SNPs and limiting to participants of European ancestry changed the estimates only minimally (< 10%; data not shown).

## Discussion

In this investigation, we identified a positive marginal association with *NOS2A* SNP rs1060826 and PD. Importantly, we also identified multiple *NOS1*–pesticide interactions, providing support for the involvement of OP pesticides in PD, especially in genetically susceptible subpopulations.

Animal models of PD suggest that environmental factors and aging together induce oxidative stress, and depending on genetic background and a biological system’s antioxidant capacities this can lead to cell death or survival (Varçin et al. 2012). Some PD-related genes might induce oxidative/nitrosative stress, such as *NOS* via regulating NO, whereas others modulate cell survival following exposure to oxidative stressors, such as *PON1* and other metabolic or antioxidant gene products. In our population, while controlling for *PON1*, OP exposure was positively associated with PD, and variation in multiple regions throughout the *NOS1* gene further modified this association. This is consistent with the hypothesis that NO and pesticides act synergistically to influence PD risk, with ROS/RNS from multiple sources acting in a potentiating manner, overwhelming the balance between pro-oxidants and the antioxidant capability of dopamine neurons.

Our population-based case–control study provided a unique opportunity to investigate *NOS* genes while adjusting for the contributions of *PON1* on OP metabolism and assess their role in modifying the effect of OP pesticide exposures in PD. Consistent with the NCI-NHGRI Working Group on Replication in Association Studies criteria for high quality replications of association results (NCI-NHGRI Working Group et al. 2007), our study provides an independent population, similarity and improvement in exposure assessment, and adequate sample size [≥ 80% power to detect previously reported marginal effect sizes, and OP interaction ORs ≥ 2.3, given summary parameters based on previous report (Hancock et al. 2008; Levecque et al. 2003)]. Additionally, we estimated pesticide exposure from multiple sources—household and agricultural uses—and the observed associations mutually corroborated each other.

PD is a commonly misdiagnosed disease (Meara et al. 1999; Wermuth et al. 2012). Different from most epidemiology studies, our PD cases were all seen and well characterized by UCLA movement disorder specialists at least once, and 70% were followed many years for disease progression (Ritz et al. 2012), minimizing bias from disease misclassification. Additionally, population controls were drawn from the same region as the cases, likely providing adequate representativeness of the source population.

The vast majority of previous epidemiologic studies investigating pesticides have relied solely on self-reported information for home pesticide use, a method prone to differential recall error, because the degree to which study participants may forget details or misreport their past pesticide use may differ between cases and controls. We improved and enriched our self-reported measure of gardening, yard, and indoor uses with the information about active ingredients provided in the CDPR’s product label database, which lists all household products registered in California for use. Thus, we depended only partially on recall for home pesticide exposure assessment: Participants did not need to report specific chemicals, but only products or types of products. In addition, we assessed ambient exposures with a GIS approach that integrates state-mandated PUR, land use data, and address information. This GIS-driven and pesticide record–based ambient exposure assessment approach does not rely on participant recall. However, our ambient pesticide exposure method does not account for factors such as wind patterns at the time of application, geographic features that may influence pesticide drift, and the assumption that the participant was at the recorded location during the relevant time period; thus, we did not eliminate the possibility of exposure misclassification. Our two exposure measures are unrelated because household use was not influenced by nearby agricultural applications; nevertheless, we saw similar patterns when assessing gene–environment interactions.

A comparison of the previously reported *NOS* SNP marginal associations is presented in Table 4 (those SNPs not included were not investigated previously). There are inconsistencies in the reported marginal associations of the *NOS* SNPs. Additionally, none of these SNP regions have emerged from PD GWA studies (Nalls et al. 2014). Although evidence for an involvement of *NOS2A* rs1060826 in PD susceptibility was relatively consistent in many candidate gene studies, there is no clear direction in the association, though a positive association similar to that seen in our population has been published before (Hancock et al. 2008). We also did not replicate previous positive marginal associations reported for *NOS1* rs1047735 or rs2682826. Gene–pesticide interactions with *NOS1* rs2682826 were first described in a study of 169 families; the authors report a positive association between ever pesticide use (in the home, garden, or work) among those with the homozygous wild-type genotype (OR = 3.52; 95% CI: 1.87, 6.95), but no association between pesticide use and PD in those with a variant allele (Hancock et al. 2008). This is in contrast to the associations we report for *NOS1* rs2682826, where we found positive associations among the OP-exposed variant carriers, with smaller or no pesticide associations in the homozygous wildtype carriers (Table 3). There may be a number of explanations for these discrepancies. For instance, marginal genetic associations ignore environmental exposures. If an environmental factor is necessary for a genetic variant to influence disease risk, populations with genetic variant carriers who are also exposed to the environmental factor are better able to detect gene–disease associations; on the other hand, not accounting for such environmental risk factors can result in varying consistency for reports of marginal associations (Ott 2004). This issue seems particularly important for the *NOS1* rs2682826, for which gene–environment interactions have been hypothesized and reported for both cigarette smoking (Levecque et al. 2003) and pesticide exposure (Hancock et al. 2008). Additionally, an inadequate reference population, disease misclassification, insufficient power, study population heterogeneity, and population stratification may also result in between-study inconsistencies (Ioannidis 2007).

**Table 4:** Comparison of SNP marginal effects from previous investigation.

Study	Population	Cases	Controls	*NOS1* rs1047735 [OR (95% CI)]	*NOS1* rs2682826 [OR (95% CI)]	*NOS2A* rs1060826 [OR (95% CI)]
Levecque et al. (2003)	French	209	488	1.20 (0.85, 1.69)^*a*^	1.53 (1.08, 2.16)^*a*^	0.50 (0.29, 0.86)^*b*^
Hague et al. (2004)	Finnish	147	137	No association (*p *= 0.63)^*c*^	No association (*p *= 0.25)^*c*^	0.50 (0.27, 0.93)^*b*^
Schulte et al. (2006)	German	340	680	NA	NA	0.89 (0.61, 1.30)^*b*^
Huerta et al. (2007)	Asturians	450	200	NA	No association^*c*^	No association^*c*^
Hancock et al. (2008)	U.S. Caucasians	169 families		Positive association, minor allele (A) over-transmitted^*d*^	Positive association, minor allele (T) over-transmitted^*d*^	Positive association, minor allele (A) over-transmitted^*d*^
NA, not applicable because not investigated. ^***a***^Dominant genetic model, CT+TT vs. CC. ^***b***^Recessive genetic model, AA vs. GG+GA. ^***c***^OR and 95% CI not provided. ^***d***^Family based transmission-disequilibrium tests were used to examine association between SNPs with PD, comparing the distributions of alleles transmitted to affected offspring to alleles not transmitted (Hancock et al. 2008). Over-transmission of the minor allele indicates the minor allele at a given locus was transmitted to those with PD more than expected, and represents a positive or “risk” association between the allele and PD.						

We found strong associations for PD in participants with certain *NOS1* genotypes exposed to commonly used OP pesticides through two independent sources—home and agricultural use—consistent with the importance of oxidative stress–inducing mechanisms in combination with increased vulnerability due to low *PON1* OP metabolizer capacity. Our findings support a role for *NOS2A* genetic variants in PD susceptibility and *NOS1* as a modifier of associations with PD in OP pesticide–exposed populations.

## Supplemental Material

(662 KB) PDF
