# Supplementary material for: Organophosphate Pesticide Exposures, Nitric Oxide Synthase Gene Variants, and Gene–Pesticide Interactions in a Case–Control Study of Parkinson’s Disease, California (USA)
Source: Environ Health Perspect. 2015 Sep 18;124(5):570–7. doi: 10.1289/ehp.1408976 (PMC4858402; doi:10.1289/ehp.1408976)
Supplement: (662 KB) PDF [file ehp.1408976.s001.acco.pdf]

Environ Health Perspect
DOI: 10.1289/ehp.1408976

Note to Readers: *EHP* strives to ensure that all journal content is accessible to all readers. However, some figures and Supplemental Material published in *EHP* articles may not conform to 508 standards due to the complexity of the information being presented. If you need assistance accessing journal content, please contact ehp508@niehs.nih.gov. Our staff will work with you to assess and meet your accessibility needs within 3 working days.

Supplemental Material

Organophosphate Pesticide Exposures, Nitric Oxide Synthase Gene Variants, and Gene–Pesticide Interactions in a Case-Control Study of Parkinson's Disease, California (USA)

Kimberly C. Paul, Janet S. Sinsheimer, Shannon L. Rhodes, Myles Cockburn, Jeff Bronstein, and Beate Ritz

Table of Contents

Table S1. Pesticide list and adjusted odds ratios.

Table S2. Interaction and joint effect estimates between *NOS1* rs2682826 and household pesticide exposure in association with PD.

Table S3. Effect estimates of *NOS1* genetic risk score (per allele) and OP exposure in association with PD.

Figure S1. Interaction between *NOS1* rs2682826 and (**A**) household OP use (excluding frequent users of non-OP pesticides) and (**B**) ambient OP exposure. The figure displays the adjusted odds ratio (OR) and 95% confidence intervals for the joint and main effect estimates of OPs and rs2682826. P-value for interaction between (**A**) household OP use and rs2682826, 0.04.and (**B**) and ambient OP exposure and rs2682826, 0.15. Adjusted for age, sex, smoking status, European ancestry, education and *PON1* metabolizing status.

Reference

**Table S1. Pesticide list and adjusted odds ratios.**					
**Pesticide**	**CDPR Chem Code / Category**	**Cases (n=306) [n (%)]**	**Controls (n=397) [n (%)]**	**Adj OR[a] (95% CI)**	**P value**
**Household Pesticides**					
Any Pesticide[b]	Occasional Use	151 (0.49)	207 (0.60)	1.00 (ref)	--
	Frequent Use	155 (0.51)	138 (0.40)	1.69 (1.21, 2.36)	0.002
OP Pesticides[b]	Occasional Use	151 (0.67)	207 (0.78)	1.00 (ref)	--
	Frequent Use	73 (0.33)	57 (0.22)	2.05 (1.30, 3.24)	0.002
**Ambient Organophosphates**					
OP Exposure Indicator[c]	>11 OPs	76 (0.25)	37 (0.09)	2.99 (1.92, 4.65)	<.0001
Monocrotophos	52	64 (0.21)	44 (0.11)	2.00 (1.29, 3.09)	0.002
Bensulide	70	5 (0.02)	5 (0.01)	1.56 (0.44, 5.49)	0.49
Dicrotophos[d]	72	5 (0.02)	2 (0.01)	--	
Trichlorfon	88	35 (0.11)	15 (0.04)	3.01 (1.58, 5.73)	0.0008
Carbophenothion	110	25 (0.08)	15 (0.04)	2.36 (1.20, 4.64)	0.01
Ddvp	187	6 (0.02)	5 (0.01)	1.81 (0.54, 6.01)	0.34
S,S,S-Tributyl Phosphorotrithioate	190	57 (0.19)	49 (0.12)	1.53 (0.99, 2.35)	0.06
Dioxathion	192	18 (0.06)	8 (0.02)	3.00 (1.26, 7.12)	0.01
Diazinon	198	97 (0.32)	106 (0.27)	1.18 (0.84, 1.67)	0.34
Dimethoate	216	137 (0.45)	99 (0.25)	2.38 (1.71, 3.33)	<.0001
Disulfoton	230	50 (0.16)	35 (0.09)	1.91 (1.19, 3.09)	0.008
Chlorpyrifos	253	62 (0.2)	71 (0.18)	1.17 (0.79, 1.73)	0.44
Ethion	268	46 (0.15)	20 (0.05)	2.94 (1.66, 5.21)	0.0002
Merphos	293	51 (0.17)	28 (0.07)	2.51 (1.52, 4.17)	0.0003
Azinphos-Methyl	314	69 (0.23)	69 (0.17)	1.34 (0.91, 1.97)	0.14
Phosmet	335	70 (0.23)	71 (0.18)	1.29 (0.88, 1.90)	0.19
Malathion	367	90 (0.29)	63 (0.16)	1.99 (1.36, 2.90)	0.0004
Oxydemeton-Methyl	382	71 (0.23)	40 (0.1)	2.58 (1.67, 3.99)	<.0001
Methyl Parathion	394	25 (0.08)	19 (0.05)	1.43 (0.74, 2.77)	0.28
Naled	418	81 (0.26)	57 (0.14)	2.08 (1.41, 3.08)	0.0002
Parathion	459	102 (0.33)	83 (0.21)	1.82 (1.28, 2.59)	0.0009
Phorate	478	58 (0.19)	43 (0.11)	1.82 (1.16, 2.84)	0.009
Phosalone	479	25 (0.08)	11 (0.03)	3.15 (1.51, 6.58)	0.002

Mevinphos	480	56 (0.18)	37 (0.09)	2.11 (1.34, 3.34)	0.001
Phosphamidon	482	11 (0.04)	5 (0.01)	3.16 (1.05, 9.44)	0.04
Sulfotep	558	5 (0.02)	9 (0.02)	0.87 (0.29, 2.64)	0.8
Demeton	566	32 (0.10)	22 (0.06)	1.90 (1.06, 3.40)	0.03
Tepp[d]	577	3 (0.01)	1 (0.003)	--	
Ethephon	1626	64 (0.21)	52 (0.13)	1.64 (1.08, 2.50)	0.02
Leptophos	1676	9 (0.03)	7 (0.02)	1.95 (0.71, 5.36)	0.19
Acephate	1685	88 (0.29)	51 (0.13)	2.57 (1.72, 3.82)	<.0001
Methidathion	1689	76 (0.25)	64 (0.16)	1.65 (1.12, 2.42)	0.01
Methamidophos	1697	36 (0.12)	28 (0.07)	1.59 (0.93, 2.73)	0.09
Dialifor	1799	20 (0.07)	7 (0.02)	3.63 (1.48, 8.94)	0.005
Fenamiphos	1857	21 (0.07)	15 (0.04)	1.65 (0.81, 3.34)	0.17
Profenofos	2042	35 (0.11)	19 (0.05)	2.37 (1.30, 4.31)	0.005

[a]Adjusted for age (continuous), sex, ever-smoked, European ancestry indicator, education, and *PON1* status (O'Leary et al. 2005) .

[b]Participants with an average frequency of household OP use per year during ages 16-<10 years prior to index age that was at or above the median average use in exposed controls were assigned to the "Frequent Use" category. Those in the "Occasional Use" category had an average frequency of use per year during ages 16-<10 years prior to index age that was below the median for any household pesticide (excluded subjects who did not frequently use OPs but frequently used other pesticides, 82 cases and 81 controls). 52 controls missing household pesticide use information.

[c]Ambient pesticide exposure, counting total number of OPs exposed to (above the median level seen in exposed controls) at both occupation and residence, from 1974 (year of CA-PUR implementation) to 10 years before diagnosis or interview. Cut point based on top quartile in exposed controls. No cases (n=306) or controls (n=397) missing ambient pesticide exposure information.

[d]No OR calculated due to small numbers

Table S2. Interaction and joint effect estimates between *NOS1* rs2682826 and household pesticide exposure in association with PD.

Pesticide Exposure	*Homozygous Wild Type (CC)*				*Variant Carrier (CT+TT)*			
	**Cases n (%)**	**Controls n (%)**	**Adj OR[a] (95% CI)**	**p value**	**Cases n (%)**	**Controls n (%)**	**Adj OR[a] (95% CI)**	**p value**
**Any Household Pesticide Use[b]**								
Occasional Use	81 (0.54)	109 (0.59)	1.00		70 (0.45)	104 (0.60)	0.88 (0.57-1.36)	0.56
Frequent Use	68 (0.46)	76 (0.41)	1.32 (0.83-2.11)	0.24	87 (0.55)	69 (0.40)	1.80 (0.15-2.84)	0.01
p value for interaction								*0.18*
**Household non-OP Pesticide Use[c]**								
Occasional Use	81 (0.69)	109 (0.75)	1.00		70 (0.60)	104 (0.69)	0.87 (0.56-1.35)	0.53
Frequent Use (non-OPs)	36 (0.31)	37 (0.25)	1.33 (0.75-2.36)	0.33	46 (0.40)	47 (0.31)	1.38 (0.82, 2.32)	0.23
p value for interaction								*0.66*

[a]Adjusted for age (continuous), sex, ever-smoked, minority status, and *PON1* status (O'Leary et al. 2005) .

[b]Participants with an average frequency of household OP use per year during ages 16-<10 years prior to index age that was at or above the median average use in exposed controls were assigned to the "Frequent Use" category. Those in the "Occasional Use" category had an average frequency of use per year during ages 16-<10 years prior to index age that was below the median for any household pesticide (excluded subjects who did not frequently use OPs but frequently used other pesticides).

[c]"Frequent Use (non-OPs)" and "Occasional Use" same as described above except those in the "Frequent Use (non-OPs)" had an average frequency use per year to any non-OP pesticides at or above the median in exposed controls (e.g excluding frequent users of OPs).

Table S3. Effect estimates of *NOS1* genetic risk score (per allele) and OP exposure in association with PD.

	Adj OR[a] (95% CL)	p value	Adj OR[a] (95% CL)	p value	p for interaction
	5 SNP *NOS1* Genetic Risk Score (GRS)				
Ambient OP Exposure	None/Low		High		
GRS (per 1 variant allele)[b]	1.04 (0.97, 1.12)	0.26	1.90 (1.04, 3.43)	0.03	0.01
Household OP Use	Occasional Use		Frequent Use		
GRS (per 1 variant allele)[b]	1.00 (0.91, 1.10)	0.98	1.45 (0.80, 2.63)	0.22	0.07
	3 SNP *NOS1* Genetic Risk Score (GRS)				
Ambient OP Exposure	None/Low		High		
GRS (per 1 variant allele)[b]	1.06 (0.95, 1.18)	0.33	2.20 (1.27, 3.80)	0.42	0.01
Household OP Use	Occasional Use		Frequent Use		
GRS (per 1 variant allele)[b]	1.00 (0.87, 1.16)	0.95	1.63 (0.93, 2.87)	0.09	0.09

[a]Adjusted for age (continuous), sex, ever-smoked, European ancestry, education, and *PON1* status (O'Leary, 2005).

[b]Range for 5 SNP GRS 0-10 variant alleles, for 3 SNP GRS 0-6 variant alleles; treated as linear variables

1-A *NOS1* rs2682826 and household OP use

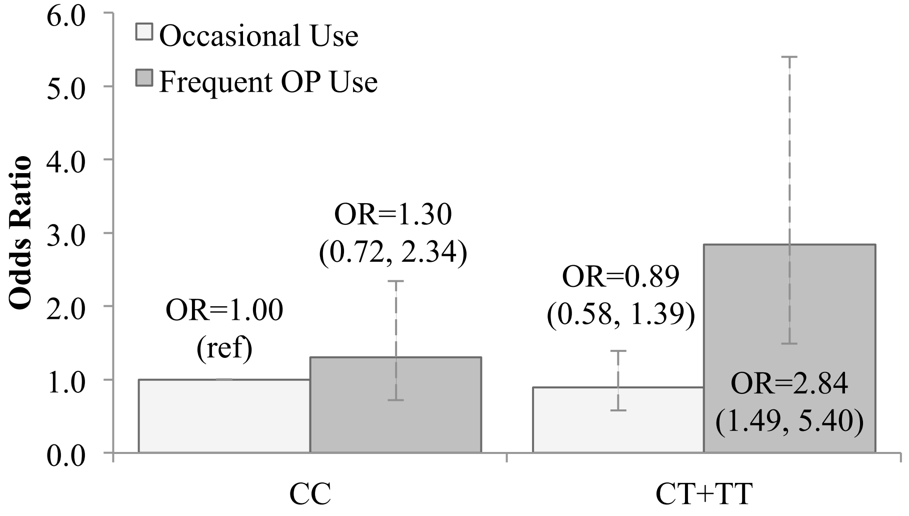

1-B *NOS1* rs2682826 and ambient OP exposure

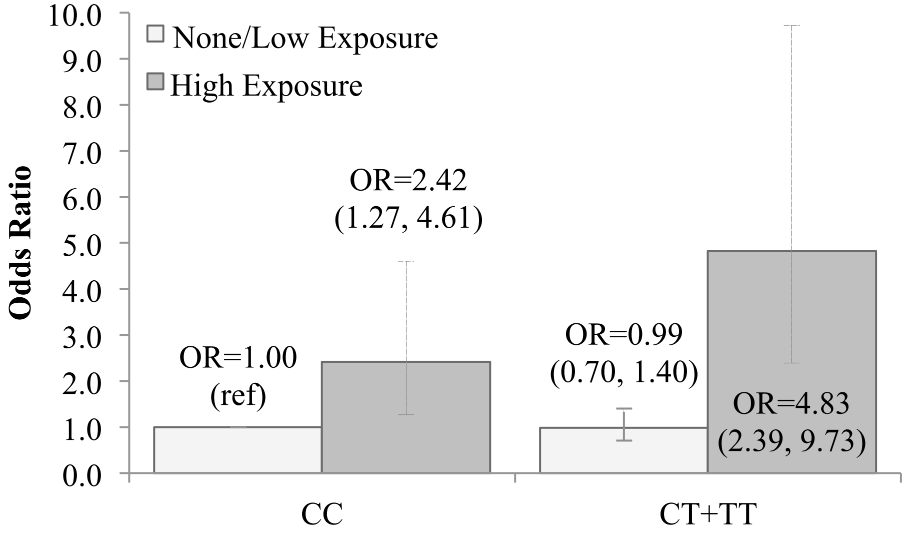

Figure S1. Interaction between *NOS1* rs2682826 and (**A**) household OP use (excluding frequent users of non-OP pesticides) and (**B**) ambient OP exposure. The figure displays the adjusted odds ratio (OR) and 95% confidence intervals for the joint and main effect estimates of OPs and rs2682826. P-value for interaction between (**A**) household OP use and rs2682826, 0.04.and (**B**) and ambient OP exposure and rs2682826, 0.15. Adjusted for age, sex, smoking status, European ancestry, education and *PON1* metabolizing status.

Reference

O'Leary KA, Edwards RJ, Town MM, Boobis AR. 2005. Genetic and other sources of variation in the activity of serum paraoxonase/diazoxonase in humans: consequences for risk from exposure to diazinon. Pharmacogenet. Genomics 15:51–60; doi:10.1097/01213011-200501000-00008.
